# Exposure to Heavy Metals in Soot Samples and Cancer Risk Assessment in Port Harcourt, Nigeria

**DOI:** 10.5696/2156-9614-9.24.191211

**Published:** 2019-11-27

**Authors:** Ihesinachi A. Kalagbor, Amalo N. Dibofori-Orji, Ozioma A. Ekpete

**Affiliations:** 1 Research & Development Centre, Kenule Beeson Saro-Wiwa Polytechnic, Bori, Nigeria; 2 Department of Chemistry, Ignatius Ajuru University of Education, Port Harcourt, Nigeria

**Keywords:** soot, illegal refining, Port Harcourt, five heavy metals, Pearson correlation, cancer risks, carcinogenicity

## Abstract

**Background.:**

Port Harcourt is an oil-rich city in Nigeria's Niger delta region. For over two years, Port Harcourt experienced black soot deposition in the environment. In November 2016, residents woke up to black soot covering cars, clothes, houses, plants, etc. Soot concentrations continued to increase until the first quarter of 2017. After public outcry, the frequency and concentration of soot deposition began to decline.

**Objective.:**

The present study was carried out to determine the presence and levels of heavy metals in soot along with a cancer risk assessment of heavy metals exposure in Port Harcourt, Nigeria.

**Method.:**

Three residential locations were sampled: Aba road, Woji and Iwofe. Sampling was performed from 6:00 am to 6:00 pm, to simulate the estimated duration that most residents who do not work in offices are exposed to soot in places such as the open market and business areas. Five heavy metals (iron (Fe), nickel (Ni), chromium (Cr), cadmium (Cd) and lead (Pb)) were investigated. The data obtained were subjected to Pearson correlation and one-way analysis of variance using SPSS software to test the correlation and significant differences between metals concentrations.

**Results.:**

Lead was found to have a significant correlation with Cd (0.808), indicating that both metals originated from the same source. Concentrations of heavy metals were higher than control values and the World Health Organization's specifications for ambient air. Chromium concentrations were the lowest. The order of concentration of heavy metals was Fe > Pb > Cd > Ni > Cr.

**Conclusions.:**

Non-carcinogenic and carcinogenic health risks of these heavy metals were evaluated using the target hazard quotient (THQ) and the incremental lifetime cancer risk (ILCR). Obtained ILCR values were within the acceptable limits for cancer risks. However, the total ILCR values for Cd and Pb for children were 3 times higher than those for adults. This is a source of concern as their prevalence in ambient air puts children and residents in Port Harcourt metropolis at risk of various types of cancers.

**Competing Interests.:**

The authors declare no competing financial interests.

## Introduction

The atmosphere consists of gaseous and non-gaseous substances, present in the form of solid or liquid particles dispersed into the air as aerosols or particulate matter and synonymous with air pollution.^1^,^2^ Large amounts of smoke and other forms of gaseous waste released into the air are a source of unhealthy ambient air. Pollutants are released faster than they can be absorbed and dispersed by the atmosphere.^3^ Several heavy metals have been determined in air samples across Europe, South America, Russia, Africa and Asia.^4–12^ Concentrations of heavy metals in the air are influenced by the speed and direction of the wind and seasonal variation.^9^ During dry periods and the harmattan season, the air is often laden with high levels of dust and particulate matters, especially in the Sahara and Sub-Saharan regions of Africa. The northeast trade wind carries particulate matter along its path, and during outdoor activities, people are at risk of coming into contact with airborne metals.^13^ Heavy metals in environmental dust can enter human tissues and internal organs; they accumulate inside the human body through respiration, inhalation, skin contact, ingestion and absorption.^14–18^ Many reviews have been published regarding heavy metals in ambient air.^2^,^10^,^19^,^20^ Major air pollution sources have been identified as coal burning, vehicular emissions and global industrial activities.^21–25^ Particulate heavy metals can have severe toxic and carcinogenic effects on humans when inhaled in high concentrations.^26^ The International Agency for Research on Cancer (IARC) has classified arsenic (As), cadmium (Cd), chromium (Cr) (VI), metallic nickel (Ni) and their compounds as group 1 carcinogens. Exposure to these heavy metals are associated with cancers of the lungs, liver, nose and kidney.^27^ Lead (Pb) and mercury (Hg) are listed as group 2A, 2B or 3 depending on their metallic state and compounds. Most studies have reported the major sources of particulate matter and heavy metals to be vehicular and industrial emissions and heavy metal concentrations are higher in developing countries than developed countries.^19^,^22–25^ The distribution of coarse and fine particulate matter fractions in an industrial area in Lagos State, Nigeria was investigated during the wet and dry season and the results showed that six metals (Cr, manganese (Mn), iron (Fe), copper (Cu), zinc (Zn) and Pb) had higher concentrations during the dry season.^28^ Other studies in other parts of the country and reviews confirm high concentrations of heavy metals in ambient air, especially during the dry seasons.^20^,^29–33^ The black soot in Port Harcourt was first observed in November 2016 and was reported in the Punch newspaper and by Sweet Crude reports.^34^,^35^ Related studies showed that the prevalence of lung and skin cancers were higher in Port Harcourt than Ibadan.^36^ In a similar study, it was reported that the air quality index (AQI) in certain areas of Port Harcourt posed serious health risks to individuals, especially the elderly and children who spent long hours outdoors.^37^ It therefore follows that ingestion (oral) exposure to contaminants should not be the only criteria for assessing health risks. Other routes of human exposure such as gaseous intake (inhalation) and dermal deposition should be considered.^38^ The present study was aimed at determining the presence and levels of some heavy metals in soot air samples from Port Harcourt; an oil-rich city in Nigeria's Niger delta region. For over two years, Port Harcourt has been experiencing black soot deposition. The persistent black soot began to be observed in Port Harcourt and its environs in the last quarter of 2016 and has become an environmental issue for residents of the city, state and country. The increased presence of soot in the environment is suspected to come from illegal refining around Port Harcourt City. This illegal refining is also known as artisanal refining, where crude oil is taken unofficially (most often by theft) by breaking pipelines. The crude oil is collected and then heated in large drums to distill the various fractions. These refining activities are hard to predict, as most take place at night and vary from day to day. Over 40 illicit refineries were identified in Port Harcourt during this period. During this time, law enforcement agents seized and burned the refineries and tankers carrying the illegal crude.^39–42^ This act contributed to the emission of soot and is a major cause of air pollution and environmental degradation. Lack of employment and poverty are the major reasons for illegal refining of crude oil, as the products are sold to consumers at relatively cheap rates. Several studies are ongoing to evaluate the health implications of the deposition of black soot in Port Harcourt City and its environs. The present study was carried out to determine the presence and levels of heavy metals in the soot as well as to perform a cancer risk assessment of heavy metals exposure.

**Abbreviations**
*BW*: Body weight
*CDI*: Chronic daily intake
*CSF*: Cancer slope factor
*HRI*: Hazard risk index
*ILCR*: Incremental lifetime cancer risk
*THQ*: Target hazard quotient
*USEPA*: United States Environmental Protection Agency

## Methods

All the chemicals and reagents used in the present study were of analytical grade from BDH Chemicals Ltd. (Poole, Dorset, UK). Many different methods have been used for the determination of heavy metals in ambient air, including inductively coupled plasma-optical emission spectrometry, atomic emission spectrometry, inductively coupled plasma mass spectrometry, and mass spectrometry. Only a few studies have used proton-induced X-ray emission, X-ray fluorescence, flame atomic absorption spectrometry and electrothermal atomic absorption spectrometry.

Inductively coupled plasma techniques are considered to be the best methods due to their sensitivity, reproducibility, wide dynamic concentration range and excellent detection limit.^43^ However, they are quite expensive, and operating costs are very high. Atomic absorption spectroscopy is capable of detecting a high number of elements with good detection limits and low operating costs.^13^

### Sampling

Outdoor sampling was performed every day for 12 weeks from 6:00 hours to 18:00 hours using pre-weighed 5-g sterile sheets of pure white fabric measuring 50 cm^2^. Sampling was preformed to simulate the estimated duration that most residents who do not work in offices spend in the open environment, especially in the open market and other public places. Each week, samples were collected in quadruplicates from 3 residential locations at Aba Road, Woji and Iwofe in Port Harcourt metropolis. For the sampling, plastic tongs were used to transfer the fabrics from the desiccators, where they were stored, to the respective sampling sites. At the end of the sampling period, each sample was put into labeled desiccators. These were then transported to the laboratory for digestion.

### Wet digestion

The digestion of the sample was carried out using aqua regia, a mixture of nitric acid and hydrochloric acid in the ratio 1:3.^44^ Aqua regia is considered adequate for environmental samples.^45^,^46^ The fabric samples (50 cm^2^) and the blanks (unexposed fabric cut into 50 cm^2^ lengths) were put in separate 100 ml beakers with 40 ml of the digestive mixture added to each beaker. These were placed in heating mantles and heated at 120°C for 1.5 hours. The samples were stirred continuously with glass rods to ensure even digestion. The solutions were cooled at room temperature, filtered with Whatman 44 filter paper, then transferred into 100 ml volumetric flasks. The resultant solutions were diluted to mark with deionized water.

### Heavy metals analysis

The heavy metals (Fe, Ni, Cr, Cd, and Pb) were analyzed using PerkinElmer AAnalyst 200 AA Model with an air/acetylene flame at wavelengths of 248.3 mm, 232.0 mm, 357.9 mm, 228.8 mm and 283.3 mm for Fe, Ni, Cr, Cd and Pb, respectively.

### Statistical analysis

Data analyses were carried out using Pearson correlation matrix and one-way analysis of variance. Differences between means were obtained using the least significance difference at p < 0.05.

### Cancer risk assessment

The possibility of cancer risks from soot in the air with respect to the carcinogenic heavy metals Pb and Cd were estimated using the incremental lifetime cancer risk (ILCR) *(Equation1)*.

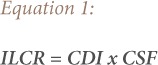
where, CDI is the chronic daily intake of carcinogen (mg/kg); and CSF is the cancer slope factor *(Equation 2)*.

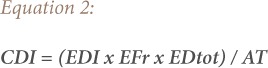
where, EDI is the estimated daily intake; EFr is the exposure frequency (365 days/year); EDtot is the exposure duration of 55 years (average lifetime for a Nigerian); and AT is the average exposure time *(Equation 3)*.

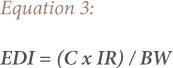
where, C is the concentration of metal; IR is the ingestion rate; BW is the average adult body weight (60.7 kg) and average child body weight (20.5 kg).

To evaluate the potential risk to human health through more than one heavy metal, the chronic hazard index (HI) was obtained as a sum of all of the target hazard quotients (THQ). The target hazard quotient is evaluated using Equation 4.

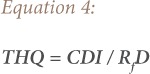
where, CDI is the chronic daily intake of a carcinogen (mg/kg); and R_f_D is the reference oral dose.

#### Cancer risks

Cancer risks associated with exposure to contaminants and heavy metals can be estimated using the ILCR. This is the incremental probability of an individual developing any type of cancer over a lifetime and is presented in Equation 1.^47^ The ILCR is obtained using the cancer slope factor (CSF), which is the risk produced by a lifetime average dose of 1 mgkg^−1^ body weight (BW) day^−1^ and is contaminant specific.^48^ The chronic daily intake of a chemical (CDI) represents the lifetime average daily dose of exposure to the chemical. Its unit is mgkg^−1^BWday^−1^ and was calculated using Equation 2. The total cancer risk from exposure to the heavy metals can be estimated using the sum of the individual metals ILCR *(Equation 5)*.

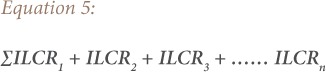
where n = 1, 2, 3 ….. is the individual carcinogenic metals (Ni, Cr, Cd and Pb). The acceptable level for cancer risk (ILCR) is considered to be within 1.0E-6 to 1.0E-4. Thus heavy metals with risk factors less than 1.0E-6 are not considered to pose a risk.

Estimated daily intake is based on BW and differs depending on the concentration of a metal in the body. The average body weight for adults is 60.7 kg and 20.5 kg for children.^49^ The estimated ingestion rates for Aba road, Woji and Iwofe are 0.144 mg/m^3^, 0.149 mg/m^3^ and 0.181 mg/m^3^, respectively, taken from the results reported for particulate matter studies in Port Harcourt for the dry season due to certain levels of exposure to a potential carcinogen.^33^ The exposure frequency is 365 days/year. The CSF is the ingestion cancer slope factor. This factor is used for ILCR calculations to evaluate the lifetime probability of developing cancer. The total exposure duration to the heavy metal is 55 years, which is the average lifespan for Nigerians *(Equation 6)*.^50^

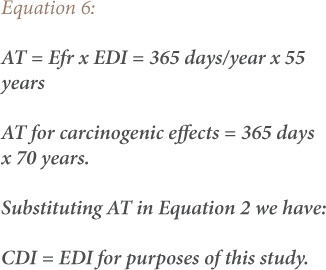
where, AT is the average exposure time for non-carcinogen in days; EFr is the exposure frequency (365 days/year); EDI is the estimated daily intake.

#### Non-carcinogenic risks

The non-carcinogenic health risk is estimated using the THQ. This parameter is used for oral exposure to heavy metals. It is defined as the ratio of the estimated daily intake to the reference oral dose and is presented in Equation 4. The reference oral dose is the daily oral exposure to a substance that will not result in any deleterious effect in a lifetime for a given population and is a useful tool in the United States Environmental Protection Agency's (US EPA) non-carcinogenic health risk assessment.^51^ The sum of the individual THQ is the health risk index = ∑THQ *(Equation 7).*

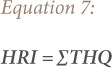



If the THQ is less than 1, the inhabitants are considered to be safe. However, if the THQ is greater than 1, the inhabitants are at risk of exposure to toxic levels of heavy metals. Although the THQ does not indicate in quantitative terms the extent to which inhabitants that are exposed to heavy metals are at risk, it does indicate levels of concern.^52^

## Results

**Figure 1:**
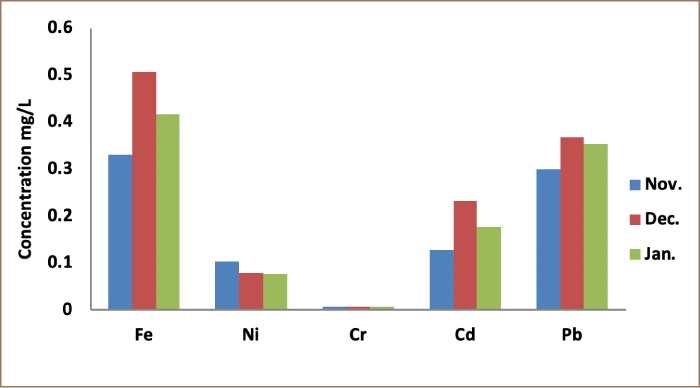
Concentrations of metals from Aba road sampling site

**Figure 2:**
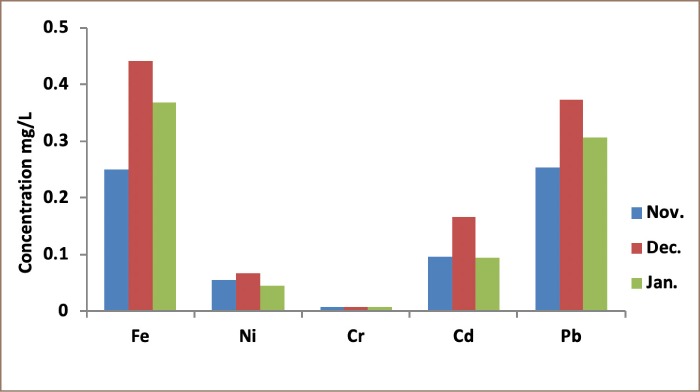
Concentrations of metals from Woji sampling site

**Figure 3:**
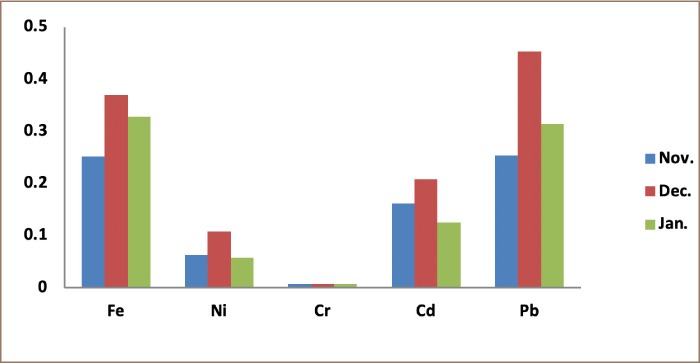
Concentrations of metals from Iwofe sampling site

Heavy metal concentrations obtained from the 3 locations, presented in Tables 1–3 and Figures 1–3, are higher than the values of the control samples. They reached their maximum values in the month of December. Aba road samples had the highest values for all of the metals; Fe (0.0506 mgkg^−1^), Ni (0.0079 mgkg^−1^) and Cd (0.0232 mgkg^−1^). Woji samples recorded Fe (0.0442 mgkg^−1^) as the only heavy metal that was higher than Iwofe samples (0.0369 mgkg^−1^). All the other metals (Ni (0.0107 mgkg^−1^) and Cd (0.0207 mgkg^−1^)) were higher in Iwofe samples than Woji samples. The concentration of Pb (0.0453 mgkg^−1^) in Iwofe was the highest recorded from the three locations. The results showed that the heavy metals in the soot air samples were highest in the Aba road samples, followed by Iwofe and then Woji. There was a statistically significant difference between the groups for the values obtained for Ni from the study sites as p = 0.025, which is less than 0.05. However, there was no significant difference for Cr, Pb, Cd and Fe for the 3 locations. These results are in agreement with the correlation studies. Lead and Cd were highly significant (p = 0.001), followed by Fe. The sum of squares for Fe was high. The levels of Fe varied from 0.0330 – 0.0506 mgkg^−1^ for Aba road samples. These values were higher than the control sample (0.029 mgkg^−1^). For the samples from Woji, Fe concentrations were 0.025 – 0.0442 mgkg^−1^, with a control sample of 0.0225 mgkg^−1^ as shown in Table 2. The concentrations of Ni were 0.0076 – 0.0103 mgkg^−1^ for Aba road, 0.0046 – 0.0067 mgkg^−1^ for Woji, and 0.0057 – 0.0107 mgkg^−1^ for Iwofe samples. These values were higher than those of the control samples of 0.0069, 0.0026 and 0.0052 mgkg^−1^, respectively. The Ni concentrations from Woji samples were the lowest. Results of the adult THQ for Ni in all 3 locations were below 1 (1.02E-3, 6.86E-4 and 1.12E-3) for Aba road, Woji and Iwofe, respectively. The concentrations of Cr in the 3 locations were the same (0.0007 mg/kg). The ILCR values obtained for adults were 8.29E-7, 8.58E-7 and 1.04E-6 for Aba road, Woji and Iwofe, respectively. Cadmium concentrations in this study were observed as follows: 0.0128–0.0232 mgkg^−1^, 0.0095–0.0167 mgkg^−1^ and 0.0125–0.0207 mgkg^−1^ for Aba road, Woji and Iwofe samples, respectively. In the present study, the Pb concentration from Aba road samples was 0.030–0.0367 mgkg^−1^, while the Pb concentration in the control sample was 0.0133 mgkg^−1^. From Woji samples, the Pb range was 0.0253–0.0373 mgkg^−1^, while the control was 0.02 mgkg^−1^. The values from Iwofe were 0.0253–0.0453 mgkg^−1^, while the value for the control was 0.0167 mgkg^−1^. The Pb ILCR values ranged from 6.48E-9 to 8.61E-9 for adults and 1.92E-8 to 2.55E-8 for children. These values are within the US EPA acceptable limits. The THQ values for Pb were 2.01E-4, 1.90E-4 and 2.53E-4 for adults from Aba road, Woji and Iwofe, respectively. The THQ values obtained for children were 5.97E-4, 5.65E-4 and 7.51E-4 for Aba road, Woji and Iwofe, respectively. The total ILCRs (∑ILCR) for children from the three locations were 3 times higher than those for adults in each location, while the HRIs were found to be 3–3.5 times higher for children than adults.

**Table 1:** Concentrations (mgkg^−1^) of Metals in Soot Samples from Aba Road Sampling Sites

**Metal**	**November**	**December**	**January**	**Mean**	**Minimum**	**Maximum**	**Control**
Fe	0.0330	0.0506	0.0416	0.0417±0.09	0.0330	0.0506	0.0290
Ni	0.0103	0.0079	0.0076	0.0086±0.01	0.0076	0.0103	0.0069
Cr	0.0007	0.0007	0.0007	0.0007	0.0007	0.0007	0.0002
Cd	0.0128	0.0232	0.0176	0.0179±0.05	0.0128	0.0232	0.0088
Pb	0.0300	0.0367	0.0353	0.034±0.04	0.0300	0.0367	0.0133

**Table 2:** Concentrations (mgkg^−1^) of Metals in Soot Samples from Woji Sampling Sites

**Metal**	**November**	**December**	**January**	**Mean**	**Minimum**	**Maximum**	**Control**
Fe	0.0250	0.0442	0.0368	0.0353±0.10	0.0250	0.0442	0.0225
Ni	0.0055	0.0067	0.0046	0.0056±0.01	0.0046	0.0067	0.0026
Cr	0.0007	0.0007	0.0007	0.0007	0.0007	0.0007	0.0002
Cd	0.0096	0.0167	0.0095	0.0120±0.04	0.0095	0.0167	0.0093
Pb	0.0253	0.0373	0.0307	0.0311±0.06	0.0253	0.0373	0.0200

**Table 3:** Concentrations (mgkg^−1^) of Metals in Soot Samples from Iwofe Sampling Sites

**Metal**	**November**	**December**	**January**	**Mean**	**Minimum**	**Maximum**	**Control**
Fe	0.0025	0.0369	0.0327	0.0316±0.06	0.0251	0.0369	0.0238
Ni	0.0062	0.0107	0.0057	0.0075±0.03	0.0057	0.0107	0.0052
Cr	0.0007	0.0007	0.0007	0.0007	0.0007	0.0007	0.0002
Cd	0.0161	0.0207	0.0125	0.0164±0.04	0.0125	0.0207	0.0080
Pb	0.0253	0.0453	0.0313	0.0340±0.10	0.0253	0.0453	0.0167

**Table 4:** Incremental Lifetime Cancer Risk for Adults and Children at Aba Road

**Metal**	**CDI (adults)**	**ILCR (adults)**	**THQ (adults)**	**CDI (children)**	**ILCR (children)**	**THQ (children)**
Fe	9.88E-5	0	1.37E-2	2.93E-4	0	4.18E-2
Ni	2.04E-7		1.02E-3	6.04E-5		3.02E-3
Cr	1.66E-6	8.29E-7	5.53E-4	4.91E-6	2.46E-6	1.64E-3
Cd	4.24E-7	2.67E-6	4.24E-4	1.26E-6	7.92E-6	1.26E-3
Pb	8.06E-7	6.85E-9	2.01E-4	2.39E-6	2.03E-8	5.97E-4

		**∑ ;=1.68E-5**	**∑ =7.78E-2**		**∑ =5.0E-5**	**∑ =2.32E-1**

**Table 5:** Incremental Lifetime Cancer Risk for Adults and Children at Woji

**Metal**	**CDI (adults)**	**ILCR (adults)**	**THQ (adults)**	**CDI (children)**	**ILCR (children)**	**THQ (children)**
Fe	8.65E-5	0	1.24E-2	2.57E-4	0	3.67E-2
Ni	1.37E-5		6.86E-4	4.07E-5		2.04E-3
Cr	1.72E-6	8.58E-7	5.72E-4	5.09E-6	2.54E-6	1.69E-3
Cd	2.94E-6	1.85E-5	2.94E-3	8.72E-6	5.50E-5	8.72E-3
Pb	7.62E-7	6.48E-9	1.90E-4	2.26E-6	1.92E-8	5.65E-4

		**∑ =1.19E-5**	**∑ =6.19E-2**		**∑ =3.5E-5**	**∑ =1.82E-1**

**Table 6:** Incremental Lifetime Cancer Risk for Adults and Children at Iwofe

**Metal**	**CDI (adults)**	**ILCR (adults)**	**THQ (adults)**	**CDI (children)**	**ILCR (children)**	**THQ (children)**
Fe	9.42E-5	0	1.35E-2	2.79E-4	0	3.99E-2
Ni	2.24E-5		1.12E-3	6.62E-5		3.31E-3
Cr	2.09E-6	1.04E-6	6.95E-4	6.18E-6	3.09E-6	2.06E-3
Cd	4.89E-7	3.08E-6	4.89E-4	1.45E-6	9.12E-6	1.45E-3
Pb	1.01E-6	8.61E-9	2.53E-4	3.00E-6	2.55E-8	7.51E-4

		**∑ =1.95E-5**	**∑ =8.95E-2**		**∑=5.77E-5**	**∑=2.65E-1**

**Table 7:** Reference Dose and Cancer Slope Factor Values (mg/kg/day)

**Metal**	**Reference dose (mg/kg/day)**	**CSF (mg/kg/day)**
Fe	0.007	0
Cr	0.003	0.5
Cd	0.001	0.38
Pb	0.004	0.0085
Zn	0.300	0
Cu	0.04	0
Ni	0.02	0

**Table 8:** One-Way Analysis of Variance

**Metal**		**Sum of squares**	**df**	**Mean square**	**F**	**Significance level**
Fe	Between groups	.068	2	.034	1.850	.199
	Within groups	.220	12	.018		
	Total	.288	14			
Ni	Between groups	.004	2	.002	5.063	.025
	Within groups	.005	12	.000		
	Total	.009	14			
Cr	Between groups	.000	2	.000	.000	1.000
	Within groups	.000	12	.000		
	Total	.000	14			
Cd	Between groups	.007	2	.003	1.605	.241
	Within groups	.025	12	.002		
	Total	.032	14			
Pb	Between groups	.000	2	.000	.012	.988
	Within groups	.103	12	.009		
	Total	.103	14			

Abbreviations: df, degrees of freedom; F, F-ratio.

**Table 9:** Pearson Correlation Matrix for the Heavy Metals in the Air Samples

		**Fe**	**Ni**	**Cr**	**Cd**	**Pb**
**Fe**	Pearson Correlation	1				
	Significance (2-tailed)					
	N	12				
**Ni**	Pearson Correlation	.455	1			
	Significance (2-tailed)	0.137				
	N	12	12			
**Cr**	Pearson Correlation	^a^	^a^			
	Significance (2-tailed)					
	N	12	12	12		
**Cd**	Pearson Correlation	.038	−.207	^a^	1	
	Significance (2-tailed)	.908	.518			
	N	12	12	12	12	
**Pb**	Pearson Correlation	−.216	−.469	^a^	.808^**^	1
	Significance (2-tailed)	.500	.124		.001	
	N	12	12	12	12	12

^**^. Correlation is significant at the 0.01 level (2-tailed).

a. Cannot be computed because at least one of the variables is constant.

Abbreviation: N, number of observations.

## Discussion

The concentrations of the heavy metals in the soot samples were highest in December. This coincides with the festival season, when the demands for petroleum products are highest. The study duration was quite short. An extended period of 6–8 months would include the rainy season, thereby providing an opportunity to capture greater variability. Chromium concentrations were the lowest and constant throughout the study period. The determination of hexavalent Cr in ambient air is often a challenge for researchers, even in speciation studies.^19^ Only a few studies have reported the determination of Cr(VI) in ambient air. The low values recorded for Cr could be attributed to the acid digestion method which reduces the recovery of hexavalent Cr from ambient air samples.^53^ Apart from Cr, all of the other metals had concentrations that were above the Food and Agriculture Organization of the World Health Organization (FAO/WHO) specified guidelines for ambient air. The concentrations of these heavy metals were high at the sampling sites. This could be attributed to the proximity of these locations to illegal crude oil refining. The order of concentration of the heavy metals was Fe > Pb > Cd > Ni > Cr.

High levels of Pb in the air have been recorded in previous studies.^8^,^9^ Exposure to high levels of Pb has more pronounced effects on children. These include behavioral changes, learning disabilities, low IQ, hearing problems, slowed growth, anemia and even death. It can cause reduced growth of the fetus in pregnant women and premature birth. High levels of Cd in the environment affect human neurological, immunological, lymphoreticular and reproductive systems^54^ and is also associated with an increased risk of renal cancer.^55^

### Correlation between heavy metals

From the results obtained in this study, presented in Figure 1, Fe had little correlation with Ni (0.455) and no correlation with Cr and Cd. There was a negative correlation between Fe and Pb. Nickel also had a negative correlation with Cd and Pb. This implies that the sources of the four metals may not have been related. However, Pb had a significant correlation with Cd (0.808), which is an indication that both metals originated from the same source. Crude oil from the Niger delta region of Nigeria contains these heavy metals as well as others. It is possible that these two metals were released in almost equal proportions as gaseous effluent in the form of soot, which is released into the air from illegal refining.

### One-way analysis of variance

From the analysis, there was no significance for the metals. However, the sum of squares was quite low for all the metals except for Fe. This is an indication that most of the values are farther apart from the mean value; hence there was a large variability in the data. However, low sums of squares were obtained for Pb, Cd and Ni, showing low variability in the set of obtained results. For Cr, the sum of squares was 0, indicating no variability.

### Cancer risk assessment

The acceptable minimum cancer risk by the US EPA ranges from 1.0E-6 to 1.0E-4. The ILCR was obtained using the CSF, which was the risk produced by a lifetime average dose of 1 mgkg^−1^ BWday^−1^ and is contaminant specific. The CSF for Pb was 0.0085 mgkg^−1^day^−1^ and 0.38 mgkg^−1^day^−1^ for Cd. The total cancer risk is expected to be a result of exposure to multiple contaminants, which is the sum of the individual metal incremental risks (∑ILCR).

### Carcinogenicity of metals in soot

Previous studies have linked exposure to soot to cancer development in humans.^27^ Leukemia, cancers of the liver and esophagus have been found to be related to exposure to soot. Our findings are supported by similar reports.^36^,^56^ The high incidence of lung and skin cancers in Port Harcourt could be the result of air pollution.

Iron is an essential macronutrient for humans. It is an integral part of hemoglobin. Deficiency of Fe results in anemia, while high levels in the body causes Parkinson's disease, Alzheimer's and Type II diabetes.^57^ Aba road samples had higher concentrations of Fe than the other locations and these values were higher than the control (0.029 mgkg^−1^). The values from Iwofe were similar to Woji samples. The range was from 0.0251 – 0.0369 mgkg^−1^. These were also higher than the control sample (0.0238 mgkg^−1^). The non-cancer risk assessment for Fe in this study was evaluated using the THQ and the HRI, as shown in Equations 4 and 7. The values obtained from THQ for Fe were all less than 1, indicating that inhabitants in these three locations are considered to be safe from the inhalation of this metal. The HRI was also less than 1. The ILCR for Fe was 0, as there is no cancer slope factor for this calculation.

Nickel is a co-factor in the Fe absorption process in the body.^58^ It is needed in the body in trace quantities for both biochemical and enzymatic reactions. The Ni concentrations from the three locations were found to range from 0.0046 – 0.0107 mgkg^−1^ with Woji samples having the lowest values. Since these values were less than 1 and the HRI was also less than 1, the inhabitants were not considered to be at risk at the time of the present study. There was no cancer slope factor for Ni, therefore its ILCR was not calculated.

Chromium occurs in six different states, but the most common oxidation states are divalent [Cr(II)], trivalent [Cr(III)] and hexavalent [Cr(VI)].^59^ Only trivalent and hexavalent chromium occur in nature. Chromium (III) is needed as a macronutrient in human bodies, occurs naturally in certain foodstuffs, and is essential for body metabolism. Chromium (VI) is ubiquitous, may cause damage to the skin and organs, and is considered to be a carcinogen. The oral reference dose for Cr(VI) as recommended by the US EPA is 3 μg/kg/day. Values beyond these levels cause health problems.^60^ The ILCR values for children from the same locations are much higher; 2.46E-6, 2.54E-6 and 3.09E-6, respectively. The THQ values were less than 1 across the 3 locations, indicating that adults and children were not at risk from exposure to Cr for the duration of this study.

Cadmium is a non-essential element for humans and is found in low concentrations in nature. Toxicity from Cd results in diseases and abnormalities of the kidneys, liver, skeleton and reproductive functions. It has been classified as a group 1 carcinogen by the International Agency for Research on Cancer. The values obtained for Cd concentrations were higher than those reported in other studies.^36^ The non-cancer risk evaluation using THQ indicated high values for children compared to the THQ values for adults. The Cd THQ values for children were 1.26E-3, 8.72E-3 and 1.45E-3 for Aba road, Woji and Iwofe, respectively. For adults, the Cd THQ values were 4.24E-4, 2.94E-3 and 4.89E-4 for the same locations, respectively. Although these values were less than 1, continued prolonged exposure may result in THQ and HRI values for Cd exposure from the soot rising to values greater than 1 (THQ, HRI>1). The Cd ILCR values for adults from the three locations ranged from 2.67E-6 to 1.85E-5. For the children, the values ranged from 7.92E-6 to 5.50E-5 and are within the acceptable limits of the US EPA.

Chronic exposure to low doses of Pb, Cd and As has been linked to development of many types of cancers.^61^ Lead is one of the many known toxic heavy metals in the environment.

## Conclusions

The results showed that after Fe, Cd and Pb had the highest concentrations in air. The order of concentration of the heavy metals was Fe > Pb > Cd > Ni > Cr.

The cumulative non-carcinogenic health risk indices from the three locations were less than 1 (HRI < 1), which implies that there was no risk associated with these heavy metals during the study period. However, even though the ILCR values were within the acceptable limits for cancer risks, the results show that the total ILCR values for Cd and Pb for children were 3 times higher than those for adults. The non-cancer risk assessment for the metals was expressed as HRI, which is the sum of the individual metals (∑THQ). These values were 3 times higher for children than for adults. The high concentrations of Pb and Cd recorded in this short – term study are a source for concern as these metals are persistent in the environment and are known to bioaccumulate. These heavy metals have been classified as carcinogens by the IARC. Their prevalence in ambient air puts the population in Port Harcourt metropolis at risk of lung, liver, blood and renal cancer, and children are at higher risk. This finding is confirmed by other studies carried out on particulate matter in polluted ambient air. Even though the HRI values were less than 1, a continuous data record would be beneficial. Further studies are needed which include the rainy season in data collection in order to capture variability over a longer time period.
